# Unraveling *Helicobacter pylori*: Insights into Pathogenesis, Immune Evasion, and Progress Toward Effective Vaccination

**DOI:** 10.3390/vaccines13070725

**Published:** 2025-07-03

**Authors:** Ayman Elbehiry, Eman Marzouk, Adil Abalkhail

**Affiliations:** Department of Public Health, College of Applied Medical Sciences, Qassim University, P.O. Box 6666, Buraydah 51452, Saudi Arabia; ar.elbehiry@qu.edu.sa (A.E.); e.marzouk@qu.edu.sa (E.M.)

**Keywords:** *Helicobacter pylori*, vaccine development, mucosal immunity, subunit vaccine, antigen delivery, public health

## Abstract

*Helicobacter pylori* (*H. pylori*) is one of the most prevalent chronic bacterial infections globally, significantly contributing to gastritis, peptic ulcers, and gastric malignancies. Its pathogenesis involves a complex array of virulence factors—including *cagA*, *vacA*, and urease—which facilitate mucosal colonization, immune evasion, and persistent inflammation. A major challenge in vaccine development is the bacterium’s ability to manipulate both innate and adaptive immune responses, resulting in limited natural clearance and long-term persistence. This review synthesizes *H. pylori* pathogenesis and host immune dynamics, highlighting their implications for vaccine design. By elucidating the molecular and cellular mechanisms underlying host–pathogen interactions, we explore how these insights inform antigen selection, adjuvant optimization, and delivery strategies. By integrating basic science with translational objectives, this review aims to support the development of an effective *H. pylori* vaccine, addressing global health needs, particularly in regions with a high infection burden and limited access to treatment.

## 1. Introduction

*Helicobacter pylori* (*H. pylori*) is one of the most prevalent chronic bacterial infections globally, colonizing the gastric mucosa of over half the population worldwide [1]. While often asymptomatic, persistent infection is a known cause of chronic gastritis, peptic ulcer disease, mucosa-associated lymphoid tissue (MALT) lymphoma, and non-cardia gastric cancer. The International Agency for Research on Cancer (IARC) consistently evaluates biological and environmental carcinogens and has recently emphasized the need to identify and mitigate infectious and dietary risk factors for cancer [2].

Despite the substantial global burden of *H. pylori* infection, no licensed vaccine is currently available. Treatment continues to rely on multidrug antibiotic regimens, which are increasingly ineffective due to rising antimicrobial resistance (AMR) [3]. Resistance to essential antibiotics—clarithromycin, metronidazole, and levofloxacin—has significantly reduced eradication rates and limited therapeutic options worldwide [4,5]. According to SalahiNiri et al. [6], mean global resistance rates are 32.6% for clarithromycin, 35.3% for metronidazole, and 13.2% for levofloxacin, exhibiting considerable regional variation. In Europe and the United States, clarithromycin resistance exceeds 20%, while metronidazole resistance surpasses 70% [7].

In Arab countries, recent systematic analyses have highlighted a concerning increase in the prevalence of the *mcr* gene and other resistance determinants in both clinical and community isolates of *H. pylori*, threatening the effectiveness of last-resort antibiotics such as colistin [8]. Bakleh et al. [8] also documented significant regional variability in AMR gene dissemination across Arab nations, emphasizing the urgent need for enhanced antimicrobial stewardship and the development of alternative preventive measures. These escalating AMR trends contribute to persistent infection and increased rates of treatment failure and recurrence, particularly in high-burden regions where diagnostic capacity is limited [6,7]. The growing challenge of multidrug-resistant *H. pylori* underscores the critical need for an effective vaccine to reduce global dependence on antibiotics and mitigate long-term complications, including gastric malignancies.

The development of an *H. pylori* vaccine remains exceptionally challenging. *H. pylori*’s ability to evade host immunity allows it to persist long-term in the stomach’s harsh acidic environment. The bacterium actively suppresses immune responses, manipulates host signaling pathways, and avoids clearance through mechanisms such as antigenic variation, biofilm formation, and the modulation of T-cell responses [9,10]. Furthermore, natural infection rarely leads to sterilizing immunity, complicating the identification of correlates of protection. Additionally, substantial genetic heterogeneity, coupled with the difficulty of generating strong, durable mucosal immune responses, continues to hinder effective vaccine design.

Despite persistent challenges, recent scientific advances have reinvigorated research into *H. pylori* vaccines. Notably, Tu et al. [11] utilized an artificial intelligence-driven framework to identify conserved, immunodominant multiepitope sequences across diverse *H. pylori* strains. The in silico-designed vaccine construct demonstrated high antigenic affinity and favorable immune simulation profiles, offering a promising foundation for next-generation, precision-guided vaccine development. Simultaneously, Yunle et al. [12] provided a comprehensive review of emerging antigen candidates, including urease, *vacA*, and *oipA*, and highlighted the potential of novel adjuvants such as CpG oligodeoxynucleotides and cholera toxin B subunit derivatives. Their analysis further emphasized the importance of mucosal delivery strategies, particularly oral and intranasal routes, for eliciting robust local immune responses within the gastric mucosa.

These innovations, which encompass immunogen design, adjuvant selection, and advanced delivery platforms, collectively offer a renewed and realistic opportunity to overcome longstanding barriers in *H. pylori* vaccine development. As global health initiatives increasingly prioritize antimicrobial stewardship and cancer prevention, developing a safe and effective *H. pylori* vaccine remains a key scientific priority and a pressing public health need.

This review offers a comprehensive overview of the current vaccine platforms under investigation, which include subunit, DNA-based, live-attenuated, vector-based, epitope-based, and virus-like particle vaccines. Additionally, it explores promising mucosal adjuvants and innovative delivery systems. The review also discusses significant translational challenges, such as immune evasion, strain variability, and mucosal delivery barriers. Furthermore, it highlights emerging technologies relevant to *H. pylori* vaccine development, including AI-guided antigen selection, nanotechnology-based delivery systems, and advances in mucosal immunology.

## 2. Biology and Clinical Relevance of *H. pylori*

### 2.1. Microbiology and Structure of H. pylori

*H. pylori* is a Gram-negative, microaerophilic, spiral-shaped bacterium equipped with multiple unipolar flagella that facilitate motility within viscous gastric mucus [13]. Its helical shape enhances mucus penetration, enabling effective navigation toward the gastric epithelium rather than direct tissue invasion [14]. Acid resistance is primarily mediated by urease activity, which hydrolyses urea into ammonia and carbon dioxide, buffering the acidic environment and enabling bacterial survival in the stomach [15]. The outer membrane proteins (OMPs) of *H. pylori*, such as *babA*, *sabA*, and *oipA*, are essential for adhesion and colonization of the gastric epithelium [16]. Additionally, the cytotoxin-associated gene pathogenicity island (*cagPAI*), particularly the *cagA* gene, encodes a type IV secretion system (T4SS) that translocates the *cagA* protein into host epithelial cells, leading to aberrant signaling and inflammation [17]. Recent proteomic studies have revealed that more than 60 OMPs are involved in adhesion, immune evasion, and nutrient uptake, reflecting *H. pylori*’s complex interactions with the host [18]. Furthermore, electron microscopy has shown that its peptidoglycan structure supports its spiral morphology, potentially influencing host immune detection [19].

In addition to its interactions with host epithelial surfaces, *H. pylori* competes with the native gastric microbiota to establish a persistent niche. The stomach was once considered sterile, but it is now recognized to harbor a unique, although low-diversity, microbial community that contributes to mucosal homeostasis and pathogen resistance [20,21]. To overcome colonization resistance, *H. pylori* employs several mechanisms, including altering the gastric pH through urease activity, disrupting tight junctions, and producing antimicrobial peptides that suppress commensal competitors [22]. Moreover, *H. pylori* modifies mucosal glycan expression to selectively adhere to and outcompete resident microbes, a strategy that facilitates niche dominance [23].

**Figure 1:**
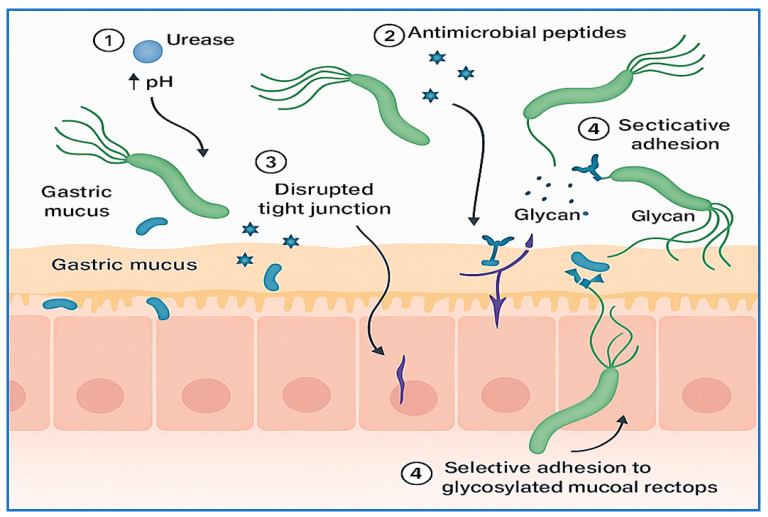
Mechanisms of gastric microbiota displacement by *H. pylori*. This conceptual illustration depicts how H. pylori establishes colonization by competing with the native gastric microbiota. Key mechanisms include (1) urease-mediated alkalinization of the gastric environment, which reduces acid-dependent microbial competitors; (2) secretion of antimicrobial peptides targeting commensals; (3) disruption of epithelial tight junctions to facilitate mucosal infiltration; and (4) selective adhesion via glycan-binding adhesins to displace resident microbes. These strategies collectively promote gastric dysbiosis, enabling chronic infection and impairing immune priming, with potential consequences for host–pathogen interactions and vaccine efficacy.

The disruption of microbial equilibrium not only favors *H. pylori* persistence but also may contribute to gastric dysbiosis, with implications for disease progression and vaccine efficacy. Understanding these microbial dynamics is essential for developing targeted interventions that promote both microbial balance and effective immune priming. The key mechanisms underlying this competitive displacement are summarized in Figure 1, which illustrates how *H. pylori* modifies its environment and disrupts the microbial ecosystem to secure a colonization niche.

### 2.2. Colonization and Survival Mechanisms

**Figure 2:**
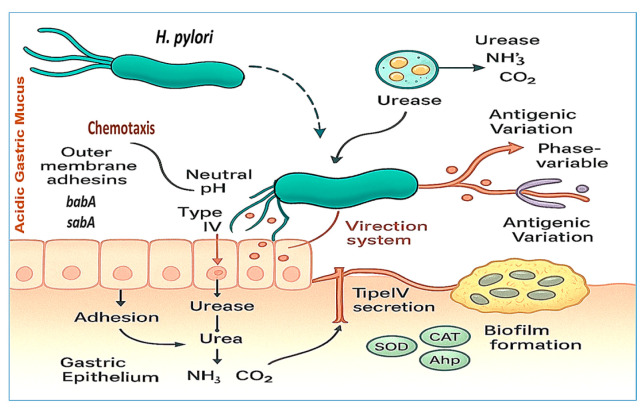
*H. pylori* strategies for colonization and survival in the gastric environment. This figure illustrates key virulence factors, including the helical morphology and unipolar flagella, which facilitate motility through gastric mucus and chemotaxis toward urea and pH gradients. Acid neutralization is achieved through urease activity, which converts urea into ammonia and carbon dioxide. Outer membrane adhesins (e.g., babA and sabA) mediate adherence to the gastric epithelium, whereas the T4SS delivers effector proteins such as cagA into host cells, promoting inflammation and altering signaling pathways. Additional features include biofilm formation and antigenic variation, both of which contribute to immune evasion and persistent colonization.

Once *H. pylori* enters the host, it must overcome multiple physical and biochemical barriers to colonize the gastric mucosa. Its spiral morphology and sheathed flagella enable efficient motility through viscous gastric mucus, guided by chemotactic responses to pH and urea gradients, which direct the bacterium toward the relatively neutral environment near the epithelial surface [24]. Upon reaching the gastric epithelium, *H. pylori* employs a repertoire of outer membrane adhesins—including *babA*, *sabA*, and *hopQ*—to bind specific host glycan structures and CEACAM receptors. This attachment not only anchors the organism securely within the gastric niche but also facilitates the translocation of virulence factors such as *cagA* into host cells via the type IV secretion system [25]. Figure 2 provides a schematic overview of these colonization and survival mechanisms, including motility, adhesion, acid neutralization, antioxidant defenses, biofilm formation, and antigenic variation.

A pivotal adaptation that enables *H. pylori* survival in the gastric environment is its production of urease, a nickel-dependent enzyme that hydrolyzes urea into ammonia and carbon dioxide. This biochemical reaction buffers the surrounding pH, effectively neutralizing gastric acid and protecting the bacterium from acid-mediated damage [26]. Additionally, *H. pylori* employs antioxidant defenses—such as superoxide dismutase, catalase, and alkyl hydroperoxide reductase—to detoxify reactive oxygen species generated by host immune cells [27]. Outer membrane vesicles (OMVs) have also emerged as significant contributors to *H. pylori* colonization and persistence. These vesicles encapsulate virulence factors—including *vacA*, urease, and adhesins—and are capable of modulating host cell signaling, promoting immune evasion, and enhancing biofilm development [28].

Furthermore, *H. pylori* forms biofilms—structured microbial communities embedded in a protective extracellular matrix—that shield the bacterium from host defenses and increase resistance to antibiotic therapies. Biofilm-associated cells exhibit reduced metabolic activity and altered gene expression patterns, rendering them more tolerant to treatment [29]. In addition to these physical defenses, *H. pylori* displays antigenic variation through phase-variable expression of OMPs such as *oipA* and *hopZ*. This reversible switch enables immune evasion by modulating the bacterial surface architecture in response to environmental pressures [30]. Further evidence suggests that OMVs actively contribute to biofilm development by transporting structural and regulatory molecules, thereby reinforcing *H. pylori*’s ability to persist long term within the human stomach [31].

### 2.3. Host Interaction and Inflammatory Pathways

The interaction between *H. pylori* and the gastric epithelium elicits a complex and dynamic inflammatory response, which is central to both the pathogenesis of gastric diseases and the persistence of infection. Upon colonization, *H. pylori* activates host pattern recognition receptors, such as toll-like receptors (TLR2, TLR4, and TLR9), initiating downstream signaling cascades via the NF-κB and MAPK pathways. This activation results in the production of pro-inflammatory mediators, including interleukin-8 (IL-8), tumor necrosis factor-alpha (TNF-α), and interleukin-1 beta (IL-1β) [32,33]. IL-8, in particular, serves as a potent chemoattractant for neutrophils and plays a central role in the chronic inflammatory milieu observed in infected gastric tissue. A hallmark of virulent *H. pylori* strains is the presence of *cagPAI*, which encodes a type IV secretion system (T4SS). This system allows the *cagA* protein to be translocated into host epithelial cells, where it becomes tyrosine-phosphorylated at EPIYA motifs by the Src and Abl kinases. Once phosphorylated, *cagA* interacts with a range of host signaling molecules, including SHP-2, leading to cytoskeletal reorganization, disruption of tight junctions, and dysregulation of cell proliferation and polarity [17]. *CagA* also modulates β-catenin signaling and promotes epithelial‒mesenchymal transition (EMT), linking chronic infection to gastric carcinogenesis [34].

In addition to *cagA*, *vacA*—present in virtually all *H. pylori* strains—plays a pivotal role in immune evasion. *VacA* forms pores in host cell membranes and targets mitochondria, inducing apoptosis in T-cells, B-cells, and epithelial cells while impairing antigen presentation by dendritic cells [35,36]. It also inhibits IL-2 signaling and T-cell activation, thereby weakening the adaptive immune response and allowing bacterial persistence [37]. The chronic inflammation induced by persistent *H. pylori* infection leads to the sustained infiltration of neutrophils, macrophages, dendritic cells, and lymphocytes into the gastric mucosa. These immune cells release reactive oxygen species, nitric oxide, and proteolytic enzymes, causing epithelial damage and amplifying the inflammatory loop [38,39]. Over time, this inflammatory microenvironment promotes gastric atrophy, intestinal metaplasia, dysplasia, and ultimately noncardia gastric cancer—particularly in individuals infected with *cagA*-positive and *vacA* s1/m1 strains [40,41].

**Figure 3:**
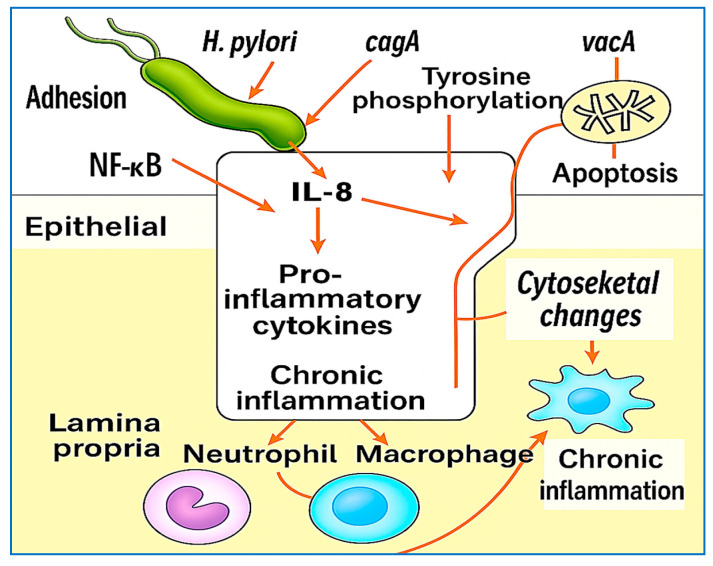
Illustration of *H. pylori*-induced host interactions and inflammatory pathways. Upon colonization, H. pylori activates toll-like receptors (TLR2, TLR4, and TLR9), triggering the NF-κB and MAPK signaling pathways and the subsequent release of pro-inflammatory cytokines (IL-8, TNF-α, and IL-1β). The cagA protein is translocated into host epithelial cells via the T4SS, where it becomes phosphorylated and interferes with SHP-2 and β-catenin signaling, promoting tight junction disruption, EMT, and carcinogenesis. VacA induces mitochondrial dysfunction, apoptosis, and immune evasion. These events collectively recruit neutrophils, macrophages, and lymphocytes; amplify oxidative damage; and sustain chronic inflammation, which can progress to gastric atrophy and cancer.

Recent transcriptomic studies have revealed that *H. pylori* modulates host gene expression through noncoding RNAs and epigenetic reprogramming, influencing immune responses, promoting epithelial cell cycle progression, and even silencing tumor suppressor genes [42,43,44]. These findings underscore the multifaceted and long-term impact of *H. pylori* on gastric mucosal immunity and host cellular homeostasis. Figure 3 provides a schematic overview of the key host–pathogen interactions involved in *H. pylori*-induced inflammation, highlighting the roles of the toll-like receptors *cagA* and *vacA* virulence factors, immune cell recruitment, and downstream signaling events that contribute to chronic inflammation and gastric carcinogenesis.

### 2.4. Associated Diseases and Clinical Outcomes

*H. pylori* infection has been extensively linked to a wide range of gastrointestinal and extra-gastrointestinal diseases, with clinical outcomes shaped by bacterial virulence factors, host immune responses, and environmental exposures [45,46,47]. Among its most serious implications is its role in noncardia gastric adenocarcinoma [48]. Chronic *H. pylori* infection initiates the Correa cascade, beginning with chronic active gastritis and progressing through stages of atrophic gastritis, intestinal metaplasia, and dysplasia, ultimately leading to gastric carcinoma [49]. This pathway has been well documented in longitudinal and mechanistic studies, and the World Health Organization (WHO) has classified *H. pylori* as a Group I carcinogen. Recent meta-analyses have confirmed that eradication of the bacterium significantly reduces the risk of gastric cancer, particularly when intervention occurs before the onset of premalignant lesions [40,50].

In addition to gastric cancer, *H. pylori* is the primary causative agent of peptic ulcer disease and is responsible for the majority of duodenal and gastric ulcers. Through mucosal inflammation, disruption of protective mechanisms, and alterations in acid secretion, bacteria promote ulcer formation. The introduction of eradication therapies has dramatically reduced recurrence rates and transformed clinical management strategies for ulcer patients [51,52]. Another important gastric condition associated with *H. pylori* infection is MALT lymphoma. In the early stages, bacterial eradication can induce complete remission in many patients, highlighting the infectious etiology of this form of gastric lymphoma and underscoring the importance of timely detection [53,54].

In functional gastrointestinal disorders, *H. pylori* also plays a role in functional dyspepsia, particularly the epigastric pain subtype. Studies have demonstrated that eradication therapy leads to meaningful symptom improvement in a subset of patients, and as a result, testing and treatment for *H. pylori* are now included in standard guidelines for managing dyspepsia [55]. Increasing evidence also supports an association between *H. pylori* infection and systemic diseases beyond the gastrointestinal tract. Iron deficiency anemia—especially in patients unresponsive to iron supplementation—has been linked to *H. pylori* through mechanisms involving chronic mucosal bleeding, hepcidin modulation, and impaired iron absorption [56,57]. Similarly, a causal relationship has been proposed between *H. pylori* and idiopathic thrombocytopenic purpura, with several studies documenting platelet count recovery following bacterial eradication [58].

Emerging literature has also explored potential links between *H. pylori* infection and extra-gastrointestinal conditions, particularly neurological, metabolic, and immunotherapy-related outcomes. However, it is important to emphasize that these associations are based primarily on observational studies and should be considered preliminary or hypothesis generating rather than conclusive. For example, epidemiological data suggest a possible association between *H. pylori* and neurodegenerative disorders such as Parkinson’s disease and Alzheimer’s disease, potentially mediated by systemic inflammation, molecular mimicry, and disruption of the gut‒brain axis [59]. Similarly, correlations have been reported between *H. pylori* infection and components of metabolic syndrome, including insulin resistance and type 2 diabetes mellitus; however, no definitive causal relationship has been established to date [60,61]. In the context of immunotherapy, early clinical data have revealed that *H. pylori* infection may negatively affect the efficacy of immune checkpoint inhibitors in patients with advanced gastric cancer. A multicenter study reported reduced progression-free and overall survival among *H. pylori*-positive individuals, possibly due to infection-mediated immune modulation [62]. While these findings are intriguing, further validation through prospective, mechanistic, and interventional research is essential before drawing firm conclusions or altering clinical practice.

**Figure 4:**
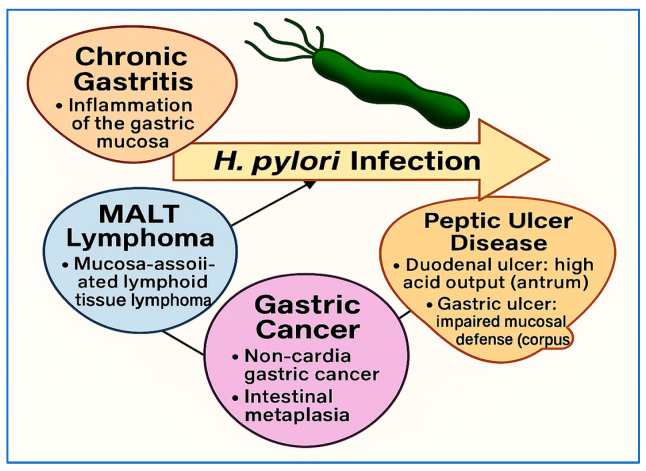
Overview of major clinical outcomes associated with *H. pylori* infection. This information summarizes key gastrointestinal conditions associated with *H. pylori* infection, including chronic gastritis, peptic ulcer disease, MALT lymphoma, and gastric cancer. It distinguishes between duodenal ulcers—typically linked to high acid output in the antrum—and gastric ulcers, which arise in the corpus due to impaired mucosal defense. The figure highlights the progression from bacterial colonization to diverse gastric pathologies.

Overall, the clinical significance of *H. pylori* infection extends well beyond the stomach. While its role in peptic ulcer disease and gastric cancer is well established, accumulating evidence highlights its contribution to hematologic, neurologic, and metabolic conditions. The growing body of literature underscores the importance of early detection, timely eradication, and a comprehensive understanding of *H. pylori*’s systemic effects. Figure 4 illustrates the major gastrointestinal outcomes associated with *H. pylori* infection, including chronic gastritis, peptic ulcer disease, MALT lymphoma, and noncardia gastric cancer. It distinguishes between duodenal ulcers—linked to high acid output in the antrum—and gastric ulcers, which arise in the corpus due to impaired mucosal defense. The figure summarizes the progression from chronic colonization to diverse pathological outcomes.

### 2.5. Epidemiology and Global Burden

*H. pylori* infection is one of the most prevalent chronic bacterial infections worldwide, affecting more than 50% of the global population [63]. Prevalence rates vary significantly by geographic region and are strongly influenced by socioeconomic, environmental, and hygiene-related factors. The highest infection rates are reported in developing countries, where limited access to clean water, inadequate sanitation, and crowded living conditions contribute to increased transmission [1]. In some regions of Africa, South America, and South Asia, the prevalence exceeds 70%, whereas rates in North America and Western Europe are generally less than 40% and continue to decline due to improved living standards and widespread antibiotic use [64].

Prevalence also varies by age, with higher rates typically observed in older individuals, reflecting cumulative exposure over time and early-life acquisition in endemic areas. Transmission occurs primarily through oral–oral and fecal–oral routes, often within households during early childhood. Key risk factors include close familial contact, poor hand hygiene, and unsafe water sources [22]. Although its prevalence remains high, its incidence (new infection rates) appears to be declining in many high-income countries as a result of improvements in public health and improved sanitation [65]. The WHO and the International Agency for Research on Cancer classify *H. pylori* as a Group I carcinogen, underscoring its established role in gastric carcinogenesis. It is estimated that *H. pylori* causes approximately 810,000 new gastric cancer cases annually, accounting for nearly 90% of all noncardia gastric cancers worldwide [65]. The population-attributable fraction (PAF) of gastric cancer caused by *H. pylori* exceeds 75% globally [66], highlighting its importance as a public health priority.

Additionally, *H. pylori* infection imposes a substantial economic burden because of the costs associated with diagnosis, eradication therapy, complications such as ulcers, and the treatment of malignancies. Despite its declining prevalence in some regions, eradication remains challenging because of increasing antibiotic resistance, inconsistent screening practices, and limited healthcare access in high-burden areas. Notably, resistance to clarithromycin and metronidazole has reduced the efficacy of standard triple therapy, prompting recommendations for region-specific treatment regimens on the basis of local resistance profiles [3,66,67,68,69,70]. Recent global strategies emphasize a multifaceted approach that combines hygiene promotion, education, early screening, and tailored eradication therapy. Targeted interventions in high-risk populations—particularly children and adults with known exposure—are essential for reducing transmission and preventing long-term complications. Given its persistence, oncogenic potential, and systemic effects, *H. pylori* remains a key priority for global infection control and cancer prevention initiatives.

### 2.6. Diagnostic Approaches for H. pylori

The accurate diagnosis of *H. pylori* infection is crucial for guiding treatment, preventing disease progression, and confirming eradication [71]. Diagnostic options are broadly classified into noninvasive and invasive tests. Among noninvasive tests, the urea breath test (UBT) is considered the most accurate, particularly for detecting active infection and for post-treatment follow-up [72]. The UBT measures labeled carbon dioxide in exhaled breath after the ingestion of urea tagged with ^13C or ^14C. Both the sensitivity and specificity exceed 90%. However, the recent use of antibiotics or proton pump inhibitors (PPIs) can reduce test accuracy; therefore, a medication-free window of at least two weeks is recommended before testing [73].

The stool antigen test (SAT) is another highly sensitive and cost-effective option that is suitable for both initial diagnosis and eradication confirmation [74]. Monoclonal antibody-based enzyme immunoassays outperform polyclonal formats. Like the UBT, the SAT accuracy is also diminished by recent antibiotic or PPI use [75]. Serological testing, typically enzyme-linked immunosorbent assay (ELISA) for *H. pylori*-specific IgG, is less clinically useful, as it cannot distinguish between active and past infections and is not appropriate for post-treatment evaluation [76].

Invasive tests, which are performed during upper gastrointestinal endoscopy, include histology, the rapid urease test (RUT), culture, and molecular diagnostics. Histology remains the gold standard for directly visualizing *H. pylori* and assessing mucosal pathology. To increase diagnostic yield, biopsy samples should be obtained from both the antrum and corpus, particularly in cases of atrophic or patchy gastritis [76]. The RUT provides rapid and inexpensive detection of urease activity in gastric tissue; however, its sensitivity is reduced in patients recently treated with antibiotics or PPIs or in cases of low bacterial density. Culture, although labor-intensive and not routinely performed, enables antimicrobial susceptibility testing—which is critical in regions with high resistance rates [3]. PCR-based assays allow for the simultaneous detection and genotyping of *H. pylori* and the identification of resistance mutations, particularly clarithromycin resistance (e.g., 23S rRNA gene mutations) [77,78]. Despite their high sensitivity, the widespread use of these assays is limited by cost and technical constraints.

Diagnostic strategies should be tailored to the clinical context, resource availability, and treatment objectives. Noninvasive tests are preferred in outpatient and follow-up settings, whereas invasive methods are reserved for endoscopic evaluations or resistance profiling. Current recommendations align with the Maastricht V/Florence Consensus Report, which provides evidence-based guidance on test selection, treatment algorithms, and antimicrobial stewardship [68]. Table 1 provides a structured comparison of the diagnostic methods used for detecting *H. pylori*, including both noninvasive and invasive techniques. The table outlines each method’s components, diagnostic accuracy, ability to detect active infection, and clinical advantages and limitations, supporting informed decision-making on the basis of patient condition, resource availability, and diagnostic goals.

### 2.7. Treatment and Management of H. pylori Infection

The eradication of *H. pylori* is a critical component of global strategies to prevent peptic ulcer disease, MALT lymphoma, and gastric cancer. The primary therapeutic goal is to achieve complete bacterial clearance, relieve symptoms, minimize recurrence, and prevent transmission. According to the Maastricht VI/Florence Consensus Report [75], eradication regimens should achieve a per-protocol success rate of ≥90%. The choice of therapy depends on several factors, including local antibiotic resistance rates, previous treatment history, patient adherence, and drug availability.

First-line treatment typically involves either bismuth-based quadruple therapy (BQT) or non-bismuth concomitant quadruple therapy. BQT combines a PPI, bismuth sub-citrate (120–240 mg four times daily), tetracycline (500 mg four times daily), and metronidazole (500 mg three or four times daily), which are administered for 10–14 days. This regimen remains effective even in regions with high clarithromycin resistance and is recommended as a first-line option in many settings [75]. Alternatively, concomitant therapy—consisting of a PPI, amoxicillin (1 g twice daily), clarithromycin (500 mg twice daily), and metronidazole (500 mg twice daily)—may be used in regions where dual resistance remains relatively low. Traditional triple therapy (PPI, clarithromycin, and amoxicillin) is no longer advised in areas where clarithromycin resistance exceeds 15% unless susceptibility testing supports its use [3,66].

Second-line therapies are typically employed after failure of initial treatment. These may include BQT (if not previously used) or levofloxacin-based triple therapy, which consists of a PPI, amoxicillin (1 g twice daily), and levofloxacin (500 mg once daily) for 10–14 days. However, increasing global resistance to fluoroquinolones has limited the utility of this approach in many regions [66]. Ideally, second-line treatment should be tailored on the basis of antimicrobial susceptibility testing, using culture or molecular diagnostics where available. For patients who have failed two prior eradication regimens, third-line or rescue therapy is recommended—again, ideally guided by susceptibility testing. In the absence of such data, the Maastricht VI Consensus Report endorses empiric use of rifabutin-based triple therapy or vonoprazan-based dual therapy [75].

Rifabutin-based triple therapy includes a PPI (standard dose twice daily), amoxicillin (1 g twice daily), and rifabutin (150 mg twice daily) for 10–14 days. This regimen may be effective even in heavily pre-treated patients, although caution is warranted because of rare but serious adverse effects such as myelotoxicity. Vonoprazan-based dual therapy has emerged as an effective alternative, particularly in areas with high clarithromycin resistance or where antibiotic stewardship is a priority. Compared with traditional PPIs, vonoprazan, a potent potassium-competitive acid blocker, offers greater stability and prolonged acid suppression. The regimen consists of vonoprazan 20 mg twice daily combined with amoxicillin 750 mg three times daily or 1 g two to three times daily, which is administered for 7–14 days. A randomized controlled trial by Furuta et al. demonstrated eradication rates as high as 94.4% with this combination, along with excellent tolerability and compliance [81].

The Maastricht VI guidelines now support vonoprazan–amoxicillin dual therapy as a valid option for first- or third-line treatment, depending on local resistance patterns and drug availability [75]. In parallel with antibiotic therapy, adjunctive strategies such as the use of probiotics have been investigated to increase treatment success and reduce side effects. Probiotics—particularly strains of *Lactobacillus*, *Bifidobacterium*, and *Saccharomyces boulardii*—have demonstrated benefits in mitigating gastrointestinal side effects (such as bloating and diarrhea) and in modestly improving eradication rates. Meta-analyses support the use of probiotics as a complementary therapy, especially in patients who experience antibiotic-associated intolerance [82,83].

In summary, the management of *H. pylori* infection should be individualized and evidence-based, taking into account regional resistance patterns, patient factors, and diagnostic access. The Maastricht VI/Florence Consensus provides comprehensive guidance on eradication strategies, emphasizing the importance of prolonged treatment duration (preferably 14 days), tailored antibiotic selection, and the integration of novel therapies such as vonoprazan-based dual regimens and third-line rifabutin-containing options. The adjunctive use of probiotics can further enhance treatment outcomes. Table 2 provides an updated comparative summary of current *H. pylori* eradication regimens, detailing components, treatment duration, intention-to-treat (ITT) and per-protocol (PP) efficacy from recent meta-analyses, resistance impact, guideline-based recommendations, and tolerability profiles. This overview facilitates evidence-based selection of therapy on the basis of patient history, local resistance data, and Maastricht VI recommendations.

### 2.8. Barriers to Vaccine Development and Translational Challenges

The development of a vaccine against *H. pylori* remains one of the most formidable challenges in modern bacterial vaccinology. Despite the pathogen’s well-established role in chronic gastritis, peptic ulcer disease, and noncardia gastric carcinoma, no licensed vaccine has yet reached clinical use. Numerous candidates have progressed to late-stage clinical trials but ultimately failed, underscoring the complex biological, immunological, technical, and translational barriers that must still be overcome.

#### 2.8.1. Immune Evasion and Mucosal Immunity Defects

A central challenge in *H. pylori* vaccine development lies in its highly evolved immune evasion mechanisms. Although infection stimulates both humoral and cellular responses, these responses are largely ineffective in clearing the bacterium, which can persist for decades within the gastric mucosa. Persistence is driven by *H. pylori*’s ability to modulate host immunity. Khamri et al. [86] reported that *H. pylori*-exposed dendritic cells acquire a tolerogenic phenotype, marked by elevated IL-10 and TGF-β, promoting the expansion of regulatory T-cells (Tregs). These Tregs suppress effector responses and permit bacterial survival while limiting mucosal damage [87,88]. In addition, *H. pylori* structurally evades innate immune recognition. Rad et al. [89] demonstrated that its hypo-acylated lipopolysaccharide (LPS) weakly stimulates TLR4, dampening pro-inflammatory signaling. Furthermore, *H. pylori* releases OMVs that exert immunomodulatory effects.

Winter et al. [90] reported that OMVs can induce both pro- and anti-inflammatory cytokines and promote the apoptosis of Jurkat T-cells, contributing to immune suppression. Despite the presence of robust IgG and IgA responses during infection, these antibodies do not confer protective immunity, as reinfection post-eradication is common. Although Th1 and Th17 responses appear to mediate protection, inducing them effectively without excessive mucosal inflammation remains a challenge [91]. The absence of validated correlates of protection further impedes rational vaccine design. Overall, *H. pylori*’s ability to manipulate antigen presentation, modulate immune activation, and exploit OMVs renders it one of the most immune-evasive bacterial pathogens, requiring novel agents.

#### 2.8.2. Antigenic Diversity and Strain Variability

The exceptional genomic plasticity of *H. pylori* presents another major obstacle to vaccine development. The bacterium displays extensive variability in key virulence factors, including *cagA*, *vacA*, *babA*, *sabA*, and *oipA*, all of which are associated with pathogenesis and immune evasion. The *vacA* gene, while universally present, presents polymorphisms in its signal (s), middle (m), and intermediate (i) regions. The *s1/m1* genotype is linked to increased cytotoxicity and inflammation, whereas the *s2/m2* strains are less pathogenic [92]. Similarly, the *cagA* gene encodes an oncoprotein delivered via a type IV secretion system and varies regionally—being present in 90% of strains in East Asia versus 50–70% in Western countries [17]. Polymorphisms in the EPIYA motif of *cagA* influence both its pathogenicity and immunogenicity. Additionally, adhesins such as *babA*, *sabA*, and *oipA* undergo phase variation, enabling immune evasion [18].

Comparative genomic analyses confirmed the rapid evolution of *H. pylori* through horizontal gene transfer and recombination. Thorell et al. [93] identified regional subpopulations with distinct virulence repertoires. This high antigenic diversity suggests that a single- or dual-antigen vaccine is unlikely to provide global protection. Although multivalent or region-specific vaccines may enhance efficacy, they introduce significant complexity in formulation, regulation, and deployment. The lack of validated immune correlates further complicates vaccine evaluation in clinical trials.

#### 2.8.3. Technical Limitations of Mucosal Delivery

Effective vaccination against *H. pylori* requires robust mucosal immunity due to its ability to colonize the stomach. Oral immunization is an attractive strategy because it mimics natural infection and can induce both mucosal and systemic responses. However, the highly acidic stomach environment and proteolytic enzymes degrade protein antigens, limiting vaccine efficacy. The gastric mucosa also lacks inductive structures such as Peyer’s patches, impeding antigen presentation and adaptive priming [94].

To overcome these barriers, various delivery technologies have been explored. Acid-resistant coatings (e.g., HP55) and biodegradable nanoparticles can shield antigens and enhance mucosal uptake, promoting IgA responses and cytokine production in animal models [95]. Solid lipid nanoparticles have delivered DNA vaccines encoding urease subunits with promising reductions in bacterial burden [96]. Liposomes also offer potential, although their stability in acidic gastric conditions remains limited [97].

Despite progress, achieving effective and durable mucosal immunity remains difficult. Inconsistent antigen uptake and poor memory responses hamper success. Sublingual immunization shows potential but has yet to be translated into effective human application [98]. A lack of safe, potent mucosal adjuvants further limits vaccine efficacy [99]. Without such adjuvants, vaccines fail to elicit protective immunity. The absence of standardized preclinical models and validated correlates of protection exacerbates these challenges [100]. Future success will likely depend on mucoadhesive carriers, acid-stable formulations, and optimized adjuvant systems.

#### 2.8.4. Translational Gaps Between Animal Models and Humans

Significant translational gaps remain between promising preclinical results and human clinical efficacy. Several key factors contribute:

##### Differences in the Immune Response

Murine and gerbil models, although valuable, do not fully capture human immune tolerance to *H. pylori*. While these models demonstrate immunogenicity and bacterial reduction, critical aspects of human immune regulation—particularly Treg-mediated tolerance—are not accurately modeled [101].

##### Genetic Diversity of *H. pylori* Strains

Preclinical models frequently utilize a limited set of *H. pylori* strains and fail to represent the global diversity in virulence profiles. This restricts the external validity of findings, as strain-specific differences significantly influence vaccine responsiveness [102].

##### Limitations of Preclinical Models

Anatomical and immunological differences between human and animal gastric mucosa reduce the predictive value of current models. Moreover, chronic infection spanning decades, as occurs in humans, is rarely replicated in animal studies [13,101].

##### Immune Modulation and Tolerance

The delicate balance between immune tolerance and inflammation in human infection, which is mediated by Tregs and other pathways, is not fully mirrored in standard animal models [100].

##### Need for Advanced Preclinical Models

Humanized mouse models, nonhuman primates, and gastric organoid systems may provide more accurate simulations of human immune responses and improve the translational potential of candidate vaccines [102].

##### Clinical Trial Discrepancies

Many vaccine candidates that show promise in animal models fail in human trials, often owing to antigenic mismatch and variability in human immune responses [103,104]. More predictive preclinical models are needed to guide vaccine development [13].

#### 2.8.5. Regulatory, Logistical, and Economic Constraints

Systemic barriers further complicate *H. pylori* vaccine development. The long latency between infection and disease makes clinical trial design challenging and increases both ethical and financial burdens [105]. Most *H. pylori*-associated morbidity occurs in LMICs, where cold chain logistics and healthcare infrastructure are limited [106]. Pharmaceutical investment has been hampered by high costs, regulatory uncertainties, and a lack of validated immune correlates [10]. However, cost-effectiveness analyses indicate that an affordable vaccine could yield substantial public health benefits in high-burden regions [106]. Achieving oral stability, mucosal immunogenicity, and broad cross-strain protection remains technically challenging [10,107].

Recent advances in immunoinformatics and pangenomic analyses have offered new avenues for the design of multivalent vaccines, although these options remain at early stages [107]. Bibliometric reviews emphasize that international collaboration, sustainable funding, and public–private partnerships are critical for progress, especially in resource-limited settings [108]. While thermostable formulations have been developed for other enteric vaccines—such as *rotavirus* (Rotasiil^®^) and *cholera* (Shanchol™)—no thermostable *H. pylori* vaccine candidates are yet available. This represents a key barrier to equitable global deployment, particularly in LMICs where maintaining cold chains is difficult [109,110].

Table 3 summarizes the major biological, technical, and translational barriers to *H. pylori* vaccine development and their implications for vaccine design, providing a framework for addressing immune evasion, antigenic diversity, mucosal delivery limitations, and implementation challenges.

### 2.9. Current Progress and Emerging Strategies in H. pylori Vaccine Development

Despite the formidable challenges outlined previously, significant progress has been made in recent years in the quest for an effective *H. pylori* vaccine. Researchers have explored a wide spectrum of vaccine platforms—including subunit, DNA, live-attenuated, vector-based, and multivalent formulations—each offering distinct immunological advantages and logistical trade-offs. Advances in genomics, bioinformatics, and mucosal immunology have further reinvigorated vaccine development, enabling rational antigen selection, optimized delivery, and enhanced immune response design.

#### 2.9.1. Subunit Vaccines

Subunit vaccines remain among the most extensively studied approaches owing to their favorable safety profiles and antigen specificity. Multiple antigens—including urease subunits (*ureA*, *ureB*), heat shock proteins (*hspA*, *hspB*), *vacA*, and *cagA*—have shown immunogenicity and protective potential in preclinical studies. Del Giudice et al. [94] demonstrated that recombinant urease B subunit immunization elicited significant protective immunity in mice. Sun et al. [111] reported that subcutaneous immunization with the *H. pylori* urease B subunit increased both systemic (serum IgG) and mucosal (gastric) anti-urease B antibodies, alongside IL-4 and IFN-γ production, indicating the ability to induce both systemic and local responses.

Similarly, Zhang et al. [112] demonstrated that intranasal immunization with *hspA* and γ-glutamyl transpeptidase (GGT), especially when combined as a fusion protein (rHspA-GGT) with the mucosal adjuvant LTB, reduced gastric bacterial loads by stimulating balanced Th1/Th2 responses. Corthésy-Theulaz et al. [113] reported that oral immunization with recombinant urease B induced protection and clearance of infection in mice. Guo et al. [114] advanced this strategy further by formulating a multivalent vaccine combining the cholera toxin B subunit with multiple *H. pylori* antigens (*ureA*, *hpaA*, *HSP60*, *ureB*), which resulted in significantly reduced colonization. While combining multiple antigens has improved protection in animal models by targeting redundant virulence mechanisms, subunit vaccines generally require potent adjuvants to achieve effective mucosal immunity—a key area of ongoing research.

#### 2.9.2. DNA and Vector-Based Vaccines

DNA vaccines encoding *H. pylori* antigens (primarily urease subunits) have been evaluated in murine models with mixed outcomes. Bégué et al. [115] demonstrated that a DNA vaccine encoding urease B induced a balanced Th1/Th2 response in mice, whereas Zavala-Spinetti et al. [116] reported limited protective efficacy, highlighting the need for further optimization. Hatzifoti et al. [117] showed that mucosal delivery of a urease B DNA vaccine could stimulate innate and cellular responses. Recent innovations have focused on enhancing DNA vaccine efficacy via novel delivery systems. Francis et al. [96] employed solid lipid nanoparticles to deliver a urease alpha subunit DNA vaccine, achieving high levels of antigen-specific antibodies and gastric CD4+ T-cell responses.

Nikzad-Chaleshtori et al. [118] used a urease E subunit-based DNA vaccine, which induced strong IgG, IFN-γ, IL-4, and IL-17 responses in mice and achieved 87.5% protection against *H. pylori* challenge. Recombinant bacterial and viral vectors (e.g., *Salmonella* and adenovirus) engineered to express *H. pylori* antigens have also been explored. Ghasemi et al. [119] developed a protective immunity-enhanced *Salmonella* vaccine vector expressing multiple antigens (*hpaA*, *Hp-nap*, *ureA*, *ureB*), which provided sterile protection in 70% of mice. Nie et al. [120] utilized an influenza A virus vector expressing *napA* and achieved robust mucosal and Th1/Th17 responses, with significant reductions in bacterial colonization.

#### 2.9.3. Live-Attenuated Vaccines

Live-attenuated bacterial vectors, particularly *Salmonella* and *Lactobacillus*, have shown promise for inducing mucosal immunity. These platforms mimic natural infection pathways and promote durable systemic and mucosal responses. Early clinical trials demonstrated safety: Angelakopoulos and Hohmann [121] reported that an attenuated *Salmonella Typhimurium* vector expressing urease was well tolerated in humans, eliciting urease-specific immunity. DiPetrillo et al. [122] and Bumann et al. [123] reported similar findings with *Salmonella* vectors expressing urease A/B subunits. Recent studies have enhanced vector performance. Ghasemi et al. [119] employed a multiantigen *Salmonella* vector that significantly reduced colonization and inflammation, whereas Nie et al. [120] demonstrated that an intranasal influenza A virus vector expressing *napA* induced potent mucosal immunity. These findings underscore the potential of multiantigen live-attenuated strategies, although further evaluation of biosafety, scalability, and regulatory hurdles is needed.

#### 2.9.4. Epitope-Based and Multiepitope Vaccines

Immunoinformatics-driven epitope-based vaccines represent a precision strategy to overcome antigenic diversity. Jebali et al. [124] designed a lipid nanoparticle-based multiepitope vaccine targeting conserved epitopes from *urease*, *cagA*, *hopE*, *sabA*, and *babA*, achieving promising immunogenicity. Cui et al. [107] and Wang et al. [125] similarly developed multiepitope vaccines targeting essential proteins, with encouraging preclinical outcomes. Urrutia-Baca et al. [126] and Ghosh et al. [127] used in silico design to develop oral multiepitope candidates with strong immunogenic profiles. While these approaches show promise, clinical validation remains necessary.

#### 2.9.5. Novel Adjuvants and Delivery Technologies

Potent adjuvants and innovative delivery platforms are essential for generating protective mucosal immunity. Jiang et al. [128] demonstrated that codelivery of *H. pylori* antigens with the cholera toxin B subunit and CpG oligodeoxynucleotides enhanced systemic and mucosal responses in mice. Hatzifoti et al. [117] also reported the synergistic effect of CpG motifs with DNA vaccines. Advanced delivery systems such as SLNs and chitosan nanoparticles have shown promise. Francis et al. [96] achieved enhanced immunity with SLN-based urease A DNA vaccines. Amaral et al. [129] demonstrated that chitosan nanoparticles carrying a multiepitope vaccine induced robust IL-17A and IFN-γ responses in mice. While these technologies offer exciting potential, further work is needed to optimize adjuvant safety and scalability for human use.

#### 2.9.6. Clinical Trial Landscape

Although many *H. pylori* vaccine candidates have shown preclinical promise, only a few have progressed to clinical trials. The most notable advancement came from a phase 3 trial in China by Zeng et al. [130], which demonstrated 71.8% efficacy in preventing infection within one year and 55% efficacy at three years using an oral recombinant urease B/heat-labile toxin B vaccine in more than 4,400 children. The vaccine was well tolerated, with no serious adverse events. Other trials, such as those by Michetti et al. [131], demonstrated the safety and immunogenicity of oral recombinant urease vaccines in adults but failed to achieve sterilizing immunity. Current limitations include the lack of correlates of protection, antigen variability among strains and challenges in achieving strong mucosal responses via noninvasive routes.

Future progress will depend on optimized antigen selection, improved delivery technologies, and standardized immunological end points. Large-scale, multinational trials are essential for evaluating vaccine generalizability and long-term efficacy. Table 4 presents a comparative overview of current *H. pylori* vaccine platforms, summarizing antigen composition, delivery routes, adjuvants, and development stages. Subcutaneous administration remains primarily used in preclinical settings with strong adjuvants, whereas the oral and intranasal routes require potent mucosal adjuvants. Live-attenuated vaccines have demonstrated safety and immunogenicity in phase I trials but lack proven human efficacy. Epitope-based approaches remain largely computational or confined to animal studies.

### 2.10. Future Perspectives and Recommendations

Despite decades of research, the development of an effective *H. pylori* vaccine remains elusive. However, the convergence of recent advances in immunology, biotechnology, and computational biology offers promising new avenues for overcoming longstanding barriers. Future efforts should prioritize several strategic directions. First, rational design approaches grounded in pangenomics, and reverse vaccinology must be expanded to identify conserved, immunogenic antigens capable of eliciting broad protection across diverse *H. pylori* strains. Particular attention should be given to antigens involved in adhesion, colonization, and immune evasion. The incorporation of multiple virulence factors into multiepitope constructs may enhance cross-protective efficacy while minimizing the risk of immune escape [126,132].

Second, effective delivery of antigens to the gastric mucosa remains a formidable challenge. Innovative carriers—including biodegradable nanoparticles, solid lipid nanoparticles, mucoadhesive hydrogels, and enteric-coated formulations—hold promise for enhancing mucosal targeting. These delivery systems should be combined with next-generation mucosal adjuvants, such as CpG oligodeoxynucleotides and cholera toxin B subunit derivatives, to induce robust local and systemic immune responses without compromising mucosal integrity [129,133].

Third, translational progress requires the adoption of sophisticated preclinical models that more accurately reflect human gastric physiology. Humanized mouse models, gastric organoids, and nonhuman primates can yield deeper insights into immunological mechanisms and correlates of protection. Harmonizing immunological endpoints between animal and clinical studies will further improve comparability and streamline regulatory approval processes [134,135].

Future clinical trials must also be rigorously designed to evaluate both short- and long-term efficacy. Greater emphasis should be placed on measuring mucosal immunity, reducing bacterial loads, and increasing the durability of immune memory. Adaptive trial designs and broader geographical representations—particularly in endemic regions—are essential for improving the robustness and global applicability of clinical outcomes [130].

Given the global burden of *H. pylori* infection and its causal link to gastric cancer, coordinated international efforts are urgently needed. Public‒private partnerships, governmental funding agencies, and global health organizations must increase *H. pylori* vaccine development as a priority within broader antimicrobial resistance and cancer prevention strategies. A comprehensive roadmap aligning scientific, clinical, and policy efforts will be critical to achieving sustainable progress [136].

Finally, successful integration of an *H. pylori* vaccine into national immunization programs will require careful planning around cost-effectiveness, target populations (e.g., school-aged children in endemic areas), delivery logistics, and health system readiness. Modeling studies are vital for supporting strategic planning and forecasting the long-term public health impact of various implementation scenarios [107]. In summary, while formidable challenges remain, the rapid evolution of molecular, immunological, and bioengineering technologies offers renewed optimism for realizing an effective *H. pylori* vaccine. This goal will demand collaborative, interdisciplinary efforts and sustained investment.

## 3. Conclusions

Although decades of scientific effort have yet to yield a licensed *H. pylori* vaccine, its development is increasingly within reach. This review highlights how the integration of modern vaccinology, systems biology, and nanotechnology is expanding the field’s possibilities. The growing array of vaccine platforms—ranging from subunit and epitope-based constructs to DNA and live-attenuated vectors—demonstrates a field that is both evolving and accelerating. Advances in mucosal delivery systems, antigen design through immunoinformatics, and next-generation adjuvants are challenging traditional vaccine paradigms. Success hinges on overcoming the translational barriers that separate promising preclinical results from meaningful clinical outcomes. Key steps include standardizing immune correlates, optimizing human-relevant models, and ensuring broad trial representation across endemic regions. An *H. pylori* vaccine must be viewed not only as a scientific achievement but also as a vital public health intervention with the potential to reduce antibiotic resistance and prevent gastric cancer in vulnerable populations. With the convergence of political will, strategic investment, and scientific innovation, the question of realizing an *H. pylori* vaccine is now a matter of timing rather than feasibility. The next phase of *H. pylori* vaccinology requires bold, cross-disciplinary collaboration, and that effort must commence immediately.

## Figures and Tables

**Table 1:** Overview of diagnostic methods for *H. pylori*: characteristics, accuracy, and clinical utility.

Diagnostic Method	Type	Detects Active Infection	Sensitivity (%)	Specificity (%)	Advantages	Limitations	Key Reference(s)
UBT	Noninvasive	Yes	90–95	95–98	High accuracy, reliable for pre- and post-treatment	Affected by PPIs, antibiotics; requires patient preparation	[75]
SAT	Noninvasive	Yes	90–94	92–97	Simple, cost-effective, suitable for screening and follow-up	Affected by PPIs, antibiotics; may have false negatives	[79,80]
IgG ELISA	Noninvasive	No	80–85	75–80	Useful in epidemiological studies, widely available	Cannot distinguish active vs past infection	[75]
Histology	Invasive	Yes	92–98	95–99	Gold standard with mucosal assessment, detects pathology	Requires endoscopy and trained histopathologist	[75]
RUT	Invasive	Yes	85–95	95–98	Rapid, inexpensive, commonly available in endoscopy	Sensitivity reduced by recent antibiotics or PPIs	[75]
Culture	Invasive	Yes	70–90	100	Allows antibiotic susceptibility testing	Technically demanding, not routinely available	[3]
PCR	Invasive	Yes	95–99	98–100	Detects resistance mutations and genotype information	Expensive, requires advanced lab infrastructure	[52]

**Table 2:** Comparative summary of *H. pylori* eradication regimens: components, duration, updated ITT and PP efficacy, resistance impact, clinical use, and recent guideline recommendations.

Regimen	Duration (Days)	Components	Efficacy (ITT/PP)	Resistance Impact	Recommended Use	Common Adverse Effects	Reference
BQT	10–14	PPI + Bismuth + Tetracycline + Metronidazole	80–85%/85–90%	Effective against clarithromycin-resistant strains	First-line in high clarithromycin resistance areas	GI upset, metallic taste	[75]
Concomitant Quadruple Therapy	10–14	PPI + Amoxicillin + Clarithromycin + Metronidazole	80–86%/85–91%	Reduced efficacy in dual resistance	First-line in low dual resistance areas	Diarrhea, taste alteration	[84]
Levofloxacin-based Triple Therapy	10–14	PPI + Amoxicillin + Levofloxacin	72–78%/78–84%	Impaired by fluoroquinolone resistance	Second-line if BQT not used	Tendonitis, GI upset	[3]
Rifabutin-based Triple Therapy	10–14	PPI + Amoxicillin + Rifabutin	76–82%/80–87%	Low cross-resistance	Third-line rescue therapy	Myelotoxicity, nausea	[75]
Vonoprazan + Amoxicillin Dual Therapy	7–14	Vonoprazan + Amoxicillin	85–90%/90–94%	Highly effective in CLA-resistant strains	First- or third-line in CLA-resistant areas	Minimal, well tolerated	[83]
BQT + Probiotics (Adjunct)	10–14	BQT + Lactobacillus/Bifidobacterium/*S. boulardii*	Modest increase (2–5%)	Adjunct only, not affected by resistance	To reduce side effects, improve compliance	Rare; mild GI symptoms	[85]

**Table 3:** Key Barriers to *H. pylori* Vaccine Development and Their Implications for Design.

Barrier Category	Description	Implications for Vaccine Design	Key References
Immune Evasion and Mucosal Immunity	*H. pylori* suppresses dendritic cells and Tregs; downregulates pro-inflammatory responses	- Enhance Th1/Th17 activation - Include mucosal adjuvants - Avoid excessive inflammation	[86,87,89,90]
Antigenic Diversity and Strain Variability	High polymorphism in virulence genes (*cagA*, *vacA*, *hpaA*) with regional diversity	- Use multivalent vaccines - Consider regionally tailored or strain-specific strategies	[13,93]
Mucosal Delivery Limitations	Low gastric pH and absence of inductive sites (MALT) hinder delivery	- Incorporate acid-resistant carriers - Use strong mucosal adjuvants (e.g., CTB, nanoparticles)	[28,105]
Translational Gaps: Animal to Human Models	Limited model reproducibility; species differences in infection clearance	- Advance human-relevant models (e.g., organotypic, gastric organoids) - Improve predictive accuracy	[101,102]
Regulatory, Logistical and Economic Constraints	Limited funding, infrastructure, and lack of surrogate endpoints	- Develop thermostable, oral, easy-to-administer vaccines - Align clinical trials with LMIC needs	[10,106,107,108]

**Table 4:** Summary of *H. pylori* vaccine platforms, status, and delivery strategies.

Vaccine Type	Key Antigens/Constructs	Delivery Route	Adjuvant	Clinical Status	Key Notes
Live-Attenuated	Urease-deficient *Salmonella* expressing *H. pylori* antigens	Oral	None or mucosal adjuvant (e.g., CTB)	Phase I completed	Preliminary safety and immunogenicity shown in Phase I trials; no conclusive protective efficacy demonstrated in humans
Inactivated Whole-Cell	Formalin-killed *H. pylori* cells	Oral	Mucosal adjuvants (e.g., LT, CTB)	Preclinical	Requires high-dose delivery and potent adjuvants for mucosal immunity
Protein Subunit	*ureB*, *vacA*, *cagA*, *napA*, *hpaA*	Subcutaneous or intramuscular	Aluminum hydroxide, MF59, or Freund’s adjuvant	Phase I/II (some completed)	Requires subcutaneous or intramuscular delivery in preclinical models with purified antigens and strong systemic adjuvants; oral mucosal delivery remains challenging; moderate immunogenicity; acceptable safety in early trials
DNA-Based	Plasmid-encoded urease, *cagA*	Intramuscular	None or CpG ODN	Preclinical	Induces systemic and mucosal immunity in animals; no human trials yet
Epitope-Based/Peptide	In silico-designed multiepitope constructs	Intranasal or oral	Mucosal adjuvants or delivery systems	Preclinical/computational	Confined to computational design or small animal studies; no human efficacy demonstrated to date; emerging strategy
Virus-like Particles (VLPs)	Recombinant *hpaA*-VLPs, *cagA*-VLPs	Intranasal or oral	Chitosan, MPLA	Preclinical	Emerging delivery strategy; favorable immunogenicity in murine models

## Data Availability

No new data were created or analyzed in this study. Data sharing is not applicable to this article.
